# Women’s experiences of postnatal distress: a qualitative study

**DOI:** 10.1186/1471-2393-14-359

**Published:** 2014-10-14

**Authors:** Rose Coates, Susan Ayers, Richard de Visser

**Affiliations:** School of Psychology, University of Sussex, Sussex, UK; Centre for Maternal and Child Health Research, School of Health Sciences, City University London, 1 Myddelton Street, London, EC1R 1UW UK

**Keywords:** Postnatal anxiety, Postnatal depression, Childbirth, Birth trauma, Breastfeeding

## Abstract

**Background:**

Women can experience a range of psychological problems after birth, including anxiety, depression and adjustment disorders. However, research has predominantly focused on depression. Qualitative work on women’s experiences of postnatal mental health problems has sampled women within particular diagnostic categories so not looked at the range of potential psychological problems. The aims of this study were to explore how women experienced and made sense of the range of emotional distress states in the first postnatal year.

**Methods:**

A qualitative study of 17 women who experienced psychological problems in the first year after having a baby. Semi-structured interviews took place in person (n =15) or on the telephone (n =2). Topics included women’s experiences of becoming distressed and their recovery. Data were analysed using Interpretative Phenomenological Analysis (IPA). Themes were developed within each interview before identifying similar themes for multiple participants across interviews, in order to retain an idiographic approach.

**Results:**

Psychological processes such as guilt, avoidance and adjustment difficulties were experienced across different types of distress. Women placed these in the context of defining moments of becoming a mother; giving birth and breastfeeding. Four superordinate themes were identified. Two concerned women’s unwanted negative emotions and difficulties adjusting to their new role. *“Living with an unwelcome beginning”* describes the way mothers’ new lives with their babies started out with unwelcome emotions, often in the context of birth and breastfeeding difficulties. All women spoke about the importance of their postnatal healthcare experiences in *“Relationships in the healthcare system”*. “*The shock of the new”* describes women’s difficulties adjusting to the demands of motherhood and women emphasised the importance of social support in *“Meeting new support needs”*.

**Conclusions:**

These findings emphasise the need for exploration of psychological processes such as distancing, guilt and self-blame across different types of emotional difficulties, as these may be viable targets for therapeutic intervention. Breastfeeding and birth trauma were key areas with which women felt they needed support with but which was not easily available.

## Background

Giving birth and having a new baby are emotive experiences, and many women are vulnerable to psychological problems during this time. Research examining psychological problems after birth has been largely quantitative and focused on major depression in the postnatal period [1, 2]. More recently, researchers have shown that anxiety symptoms and disorders are as common as depression in the postpartum period [3]. Levels of posttraumatic stress disorder (PTSD) following childbirth are also of clinical significance [4]. In this article we use the terms ‘distress’, ‘emotional distress,’ ‘emotional difficulties’ and ‘psychological problems’ interchangeably to refer to any psychological problem which impairs daily functioning. It was considered that mothers may identify more with terms such as ‘emotional distress’ and ‘emotional difficulties’ than with ‘psychological problems’ which is used in the academic discussion of such concepts. These psychological problems have a significant impact on women and their children, with evidence that depression and anxiety can lead to altered mother-infant interaction and developmental difficulties [1–3, 5–7].

Symptoms of distress particular to the postnatal period may be missed by research using general measures of distress and psychopathology that are not designed for use in postnatal populations [8, 9]. The postnatal period has unique physiological and psychological aspects such as fatigue, interrupted sleeping and the adoption of new routines such as breastfeeding. These unique aspects may affect responses on self-report measures which include items that are not appropriate for the postnatal period [10]. For example, the General Health Questionnaire GHQ [11] asks if the respondent has been able to leave the house as often as usual which may result in endorsement of a ‘symptom’ that is a normal component of new motherhood, leading to false ‘cases’ and, ultimately, the pathologising of motherhood [12].

Another issue is that self-report measures are validated against diagnostic criteria which may not be relevant to postnatal women. In a sample of mothers with unsettled infants, equal numbers of mothers (11%) were diagnosed with Generalised Anxiety Disorder (GAD) as were diagnosed with an anxiety disorder not otherwise specified (ADNOS), defined as the primary symptoms not being associated with OCD, social anxiety, specific phobias or panic disorder [13]. All of these women experienced uncontrollable worry about motherhood or their infant. It is therefore perhaps unsurprising that some postnatal women report clinically significant symptoms of both depression and anxiety [6, 14, 15] indicating that only part of a woman’s experience of distress is explored when using symptom measures of a specific disorder. As a result of these limitations in measurement, there could be a disparity between women’s lived experiences of distress in the postnatal period, whether those symptoms are problematic to women or a normal part of motherhood, and how those experiences are reported subject to current measurement practices.

Qualitative research examining women’s experiences of psychological disorders has also commonly worked within diagnostic categories of postnatal depression [PND; 16–20]; anxiety [16]; or post-traumatic stress disorder following childbirth [17, 18]. Despite focusing on different diagnostic categories, there is much overlap in findings. Beck [19, 20] conceptualised PND as a loss of former self and loss of control over one’s life, in both phenomenological and grounded theory studies of women attending a postnatal depression support group. High anxiety was also a key part of these women's experiences. Subsequent qualitative studies have identified important experiential aspects of PND such as: a sense of loss of autonomy, time, appearance, femininity, sexuality and occupational identity and feelings of loneliness, depression and panic [21]; feeling overwhelmed with their new responsibility and negative self evaluation related to being unsure about being able to meet their baby’s needs [22]; and limited social support and breastfeeding difficulties [23]. Hall [24] interviewed 10 women who had experienced postnatal depression: they commonly described their unrealistic expectations of motherhood as a key aspect in the development of depression.

There is far less qualitative research examining experiences of postnatal anxiety. Wardrop and Papaduik [16] interviewed six women for whom anxiety was the primary mental health concern in the first six months postpartum. A key theme for these women related to feeling misunderstood and alienated because their symptoms did not fit with the dominant concept of postnatal depression. In common with qualitative research on postnatal depression women spoke of a relationship between high expectations, perceived lack of competence as a mother and anxiety, loneliness and feeling overwhelmed. Lack of social support was also an important factor in experiences of anxiety.

In contrast, there is a substantial body of qualitative work on PTSD following childbirth. A meta-synthesis of ten qualitative studies of women’s perceptions and experiences of traumatic birth identified themes of feeling out of control, feeling inhumanely treated, feeling trapped with the childbirth experience, a ‘rollercoaster of emotions’, disrupted relationships and finding ways of succeeding as a mother after feeling their mothering ability had been hampered by a traumatic birth [25]. These qualitative studies also show the potential debilitating effect of traumatic births on breastfeeding [26], and the mother’s relationships with the father and the baby [18, 27].

In the research to date women were selected due to their experience of one specific type of disorder (i.e., depression, anxiety or posttraumatic stress disorder following childbirth) in line with the disorder-focus of most contemporary research. However several themes appear in qualitative reports that span anxiety, depression and postnatal post-traumatic stress disorder, particularly high expectations, feeling overwhelmed, perceived lack of competence as a mother, lack of social support and breastfeeding. This suggests that a transdiagnostic approach to postnatal distress may be useful. Such an approach could explain high comorbidity through establishing causal factors and maintaining processes across disorders, improve screening and identification of multiple types of postnatal distress, and help develop specific treatment components effective across a broad range of mental health problems [28].

Qualitative research that focuses on women’s actual experiences and conceptualisations of postnatal distress outside of diagnostic categories is therefore necessary. The key research question was to determine how women themselves conceptualise their postnatal distress and to obtain more information about what women themselves consider problematic or impairing and what help they would like with their distress. This study develops insight into the experiences of mothers who experienced postnatal distress. In contrast to previous research, the data highlight psychological processes experienced across different types of distress in the context of defining moments in becoming a mother; birth and breastfeeding.

## Methods

A sample of 17 women aged 23–42 took part in the study. Inclusion criteria were that women had given birth to a baby in the past year and experienced “emotional difficulties” at some point during this year. All except one woman were white and one was Chinese. Two had completed GCSE level education; six had completed A level education; five had a degree or higher degree; four had completed professional qualifications. Further characteristics of the sample are presented in Table 1. Although smaller sample sizes are often advocated in Interpretative Phenomenological Analysis (IPA) [29], larger samples are common (a review of 48 studies found samples ranged from 1–35 with a mean of 14 participants; [30]). We acknowledge that there is often a sacrifice of depth of analysis with larger samples [29]. However, we felt that because the current discourse of postnatal mental health largely focuses on postnatal depression, a larger sample size would be required to reflect the range of other emotional difficulties experienced in the postnatal period. The sampling strategy was opportunistic allowing the researchers to select participants on the basis of their experiential knowledge [31]. Advertisements were placed on relevant websites (e.g., local postnatal group Facebook pages), in local National Childbirth Trust (NCT) newsletters, and through instructors at relevant antenatal and postnatal classes e.g., pregnancy yoga classes and word of mouth.

**Table 1:** **Characteristics of the sample**

Participant no.	Age group	Age of baby at interview	Parity	Method of delivery	Referred/requested psychological services in 1^st^year postpartum?	Self-reported lifetime mental health problem?
**1**	23-29	2 months	3	NVD	Yes	Yes
**2**	35-39	6 months	1	Assisted -Forceps	No	No
**3**	30-34	2 months	1	NVD	No	Yes
**4**	23-29	4 months	1	Emergency caesarean	No	No
**5**	23-29	3 months	2	NVD	No	No
**6**	30-34	6 months	1	Elective caesarean	No	No
**7**	30-34	6 months	1	Emergency caesarean	Yes	Yes
**8**	23-29	8 months	1	NVD	No	Yes
**9**	30-34	11 months	1	Emergency caesarean	No	No
**10**	23-29	8 months	3	Emergency caesarean	Yes	Yes
**11**	35-39	1 month	2	Elective caesarean	No	No
**12**	23-29	11 months	1	NVD	Yes	Yes
**13**	35-39	8 months	1	Assisted - ventouse	Yes	No
**14**	35-39	12 months	2	Emergency caesarean	No	Yes
**15**	30-34	8 months	2	Elective caesarean	No	No
**16**	30-34	3 months	1	NVD	No	No
**17**	35-39	6 months	1	Emergency caesarean	Yes	No

NVD = normal vaginal delivery

All women who responded to the advertisement who met the inclusion criteria and wanted to take part were sent an information sheet, a consent form and demographics and pregnancy/birth questionnaire to complete, sign and send back should they wish to participate. All women who initially showed an interest took part; due to this sampling method there were not any women who were not included in the study. Ethical approval was obtained from the university Research Governance Committee and NCT Research Office. The information sheets, consent forms and interview questions were careful to avoid the term ‘depression’ and instead focused on ‘distress’ to be congruent with the study aims of exploring different types of emotional difficulties that women experience.

Interviews were conducted between September 2010 and February 2011 at women’s homes in the South East of England (n =15) or via telephone when women lived in other areas of England and had heard about the study through word of mouth (n =2). Present at the interview were the researcher (RC) and the mother participating in the interview, and in most cases her baby and/or other young children. Participants gave their consent for the interviews to be recorded and were made aware that they could stop the interview at any time without giving a reason. The interviews followed a semi-structured format whereby an interview schedule was used but the order in which questions were asked questions and answered could vary according to the responses of the participant. Follow-up questions were asked and the clarification of points which arose was also sought. Participants were first asked ‘Can you tell me about when you first started to feel distressed?’ Examples of further questions included:

- ‘What thoughts and feelings did you experience?’
- ‘How did you cope with your symptoms?’
- ‘What was your experience of seeking help (if you did so) during this period?’
- ‘What was your experience of being offered help?’

Participants were further encouraged to discuss any issues that they felt were relevant to their experience of distress. Interviews ranged from 22–72 minutes (Mean = 43 minutes). Various factors may help to explain variation in interview times. In the first interviews the questions were asked in a sequential manner to ensure that the entire schedule was covered, however, it became clear that a more open and flexible strategy would produce the most rich data as it would allow participants to follow their own stories and discuss further aspects that were not covered on the interview schedule but were important in their stories of distress (e.g. breastfeeding). Beyond this, although all mothers self-selected to take part in the study, some were more willing or better able to elaborate on their experiences.

The telephone interviews followed the same process, including being recorded. It is acknowledged that telephone interviews can limit the building of rapport and non-verbal communication cannot be tended to in the same way as face-to-face interviews; however they were no less rich in terms of outcome. The second telephone interview was one of the shortest interviews (25 minutes) whilst the first was 48 minutes.

### Analysis

Interviews were transcribed verbatim by RC and checked by RdV and SA. Participant numbers are used to ensure confidentiality. Data analysis was conducted according to the principles of interpretative phenomenological analysis (IPA; [29–32]): pre-identified themes were not used to guide analysis. This method was suitable for the purpose of the study as it aims to examine underlying cognitions and emotions as well as describe the participants’ experience.

The method involved using emic (insider) and etic (interpretative, outsider) positions [32]. The analysis followed a four stage process as follows. In step (1) of the analysis, transcripts were read repeatedly to identify accounts of experiences that were important to the interviewee. The emic phenomenological position employed here concerned hearing and understanding the participant’s story in their own words and keeping their experience at the centre of their account. In step (2), the etic phase, the accounts identified were re-read and pertinent sections summarized and given shorthand labels (codes) representing the researcher's interpretation. Steps (1) and (2) reflect the ‘double hermeneutic’ aspect of IPA whereby the participant interprets their own life experience and the researcher further interprets the participant’s account. Step (3) involved a shift to identifying how these codes clustered together into themes and how themes were related to each other. Interviews were coded on a case-by-case analysis and themes labelled using key words and phrases from participants where possible to retain an idiographic approach. In step (4) comparisons were made across the body of interviews to determine how prevalent themes were and how important they were to interviewees. The authors agreed on an approach to analysis prior to commencing analysis. The first author discussed emerging themes with the second and third authors to ensure that a consistent and balanced approach was applied to all four step described above. Self-reflexive application of these four steps meant that the authors gave priority to the interviewees’ accounts rather than their own personal or professional knowledge of the experiences of pregnancy, birth, and the postnatal period. The results section provides descriptions of these themes, using verbatim quotes to illustrate these interpretations.

## Results

Four major themes were identified: “Living with an unwelcome beginning” concerning mothers’ early days with their new baby; “Relationships within the healthcare system” speaks of mothers’ experiences with healthcare providers; “The shock of the new” relates the instant, permanent and challenging change to one’s life immediately after having a baby; and “Meeting new support needs” considers the types of needs mothers have to adapt to and satisfy. These themes will be discussed in turn using direct quotes to support them. Superordinate themes and the subthemes are presented in Table 2.

**Table 2:** **Themes and sub-themes identified in interviews**

Theme and subthemes	Percentage (n) of women who mentioned the theme
**Living with an unwelcome beginning**	**100% (17)**
Distancing and avoidance of emotions	65% (11)
Birth-related distress	65% (11)
Guilt and self-blame	59% (10)
Breastfeeding experiences	71% (12)
**Relationships in the healthcare system**	**100% (17)**
Uncared for in the healthcare system	82% (14)
Unknown in the healthcare system	59% (10)
Positive experiences	71% (12)
**The shock of the new**	**88% (15)**
Adjustment to the unknown	82% (14)
Overwhelming responsibility	47% (8)
Inexperience	53% (9)
**Meeting new support needs**	**100% (17)**
Needing and seeking support	65% (11)
Action to help move on	100% (17)

### Living with an unwelcome beginning

Eleven mothers described their new life with their baby as starting in a way that was not as they had hoped. This theme is characterised by a sense of feeling removed, or distant, from their day-to-day life with their baby. Some mothers acknowledged this sense of distance whilst others avoided their own negative emotions. The characterisation of distress as an overarching feeling of remoteness was explained by many women in terms of negative birth experiences that women often blamed themselves for, and which they found difficult to move on from. These negative experiences were often compounded by difficulties faced in one of the central aspects of caring for a new baby; breastfeeding. Women reflected on a subsequent feeling of having lost the important new-baby stage of their own and their baby’s life.

### Distancing and avoidance of emotions

Rather than being an easily describable phenomenon, or feeling categorically unhappy or worried, there was often an underlying feeling of something not being quite right or feeling out of character and somehow detached from their own life. The sense of feeling outside of one’s own head or body was described by *participant 1*, who could not pinpoint discrete emotions or feelings at first but felt an unusual sense of unease:

It is as if I was playing a part, going through the motions, so I was doing all the right things for him, playing with him, chatting to him, but not feeling like it was me or that it was very natural.

This highlights the idea that there are both ‘natural’ and ‘right’ ways of caring for a baby or of feeling about one’s baby, and for this participant, that an unsatisfactory comparison with these ways is related to feelings of disconnection. For *participant 8* the feeling of detachment was more pronounced and alien:

I really just felt like I was watching myself in day-to-day life and I wasn’t actually in my own body. It’s quite strange.

Feelings of detachment were also embodied in experiences of bonding that were not as desired. In recounting her immediate feelings of not connecting with her baby, *participant 1* alluded to the expectation of instant and intense love that new mothers sometimes expect to feel:

As soon as she was born I didn’t feel right, like I didn’t have a connection with her … I felt like it was someone else’s baby I was holding, it was really weird.

Furthermore, for *participant 12*, feeling better was characterised by starting to feel a connection with her baby:

I hadn’t spoken to anybody for weeks, so I was starting to ring people that week and said ‘I love her [her daughter], you know I’m having a nice time, I’m going for walks’ and it was just brilliant.

For some mothers, the feelings of distance were acknowledged, with a desire for those feelings to change, whilst others actively tried to avoid dealing with their feelings of distress in the hope that they would change in the near future or that keeping busy would keep them at bay. There was a sense of hope that life as a mother as it ‘should be' would present itself if one was able to deal with the present unwanted feelings of distance. In describing her avoidance of emotions, *participant 1* drew on her intense desire for a happy family life:

I didn’t want to admit that I had something wrong because I didn’t want things to go wrong … I thought it’s like a perfect kind of thing, a perfect family, like everything could be OK, maybe next week I’ll feel a bit better, but it didn’t feel any better the next week.

### Birth- related distress

Whilst for some mothers the sense of an unwelcome beginning related to a general feeling of distress in their new life as a mother, for others this theme was exemplified by reference to the temporal beginning of life; an unexpected or difficult birth experience. For most, a sense of disappointment that the birth had not been as they had hoped or expected led to feelings of their new life being at fault from the outset:

[Labour and birth] was just nothing like what I’d imagined so I just felt . . . like just at a disadvantage. Like I’d been thwarted all the way through and um something was taken away from me so I felt like I couldn’t really recover, to get back to square one, how I wanted to start out with this new life. (*Participant 7*)

Mothers made sense of their unmet expectations of birth in differing ways; some mothers felt unprepared, others felt that having prepared for the birth it should have been more as they would have expected. Premature, late, quick, or complex births all led to feelings of distress.

Feelings of distress around birth experiences left mothers feeling that they could not move on without some kind of resolution of the birth. Such resolution was described through needing to know fully about the birth, or being able to explore what had happened to them during birth, or through having space and sleep to ‘process’ what had happened, which was unlikely with a new baby to care for. In recounting her need to move on, *participant 17* stressed how she considered finding out the specifics of her experience to be the most important factor in this:

I don’t know what happened when he went into the Special Care. I never managed to find out, so I’m quite keen to find out exactly what happened, and I’m hoping that will just put a lid on it to be honest, and put it to bed.

Other factors they were not so able to influence could prevent women from moving on. Reminders of lost birth experiences revived early feelings of distress and other negative emotions, as *participant 8* recounted:

I had some friends who had children around the same time who had normal births, and when I heard that they’d had their babies I felt quite jealous and angry inside that everything was OK . . . I kind of felt “Why should they have everything perfect and I shouldn’t?”

### Guilt/Self-blame

Feelings of guilt about negative birth experiences were frequent, with mothers often feeling defeated through the birth not being the commonly-desired birthing without intervention. *Participant 13* expressed ambivalence about self-blame, seemingly unwilling to rationally blame herself but feeling something akin to it:

I don’t know if I was blaming…I wasn’t blaming myself but I still felt in some way a bit useless about it. I wanted to be the mummy who just did it all naturally and it was all gorgeous and the way it should be.

For some mothers the feelings of culpability were more explicit. Discussing birth experiences, mothers spoke of a separation between their mind and their body, blaming their body for opposing their will, and indicating the complex nature of interaction between physical and psychological control during birth and labour. Reflecting on her own experience as well as that of other mothers, *participant 16* described her feeling that her body let down both herself and her baby:

I’ve spoken to other new mums you know, and no matter what kind of experiences they’ve had, a lot of them mention the guilt word […] I had this guilt, and probably still do a little bit that my body let me and her down because she came so early, and you kind of have this guilt that you know, you somehow have caused your baby to suffer…

### Breastfeeding experiences

All but two women who experienced birth-related distress went on to experience difficulties with breastfeeding. The perception of having no control over their birth experience led to an attempt to regain control over childbearing via breastfeeding. A determination to breastfeed was present even if women felt that it took all their time and resources to succeed at this.

Sometimes the determination to breastfeed led to women feeling that they were engaged in a fight to succeed. *Participant 5* was ‘desperate’ to breastfeed but described it as the ‘hardest thing’ she’d ‘ever had to do.’ Many women expected breastfeeding to be either something ‘amazing’ (*participant 4*) or a process that would ‘be the most natural thing in the world’ (*participant 2*); or that ‘everyone thinks it’s really easy’ (*participant 11*). There was a feeling that the mechanics of breastfeeding were explained at antenatal classes but problem-solving of breastfeeding issues was not raised. *Participant 5* explained that mothers could be better prepared for feeding difficulties:

Everyone has feeding problems . . . sometimes you feel like you’re the only one, and I think if maybe they’re more open about the problems you can face, because no one actually tells you, “Oh your baby might not feed from you”. . . then it’s not such a shock.

Breastfeeding overshadowed all other aspects of daily life with women reporting feelings of anxiety, stress and frustration about their feeding experiences. The nature of new-born babies needing frequent feeding meant that these emotions could be experienced periodically throughout the day, as *participant 4* recounted:

I had anxiety every time I fed her - she would go to sleep and I would build up this worry about what would happen when she woke up again you know, would feeding go well? How long would it take?. . . I would find myself willing her to stay asleep for as long as possible just so that I wouldn’t have to do that again.

All women reported feeling unsupported with breastfeeding by healthcare professionals and were proactive with trying to access support, trying multiple helplines and charities as well as the NHS. When accessible, advice from helplines was no replacement for practical help:

I just got the usual spiel you know, lots of skin to skin contact, and all the stuff that I knew and I remember saying to her you know “I really need practical help to tell me whether we’re doing it right” and she was like ‘well, that’s the health visitor really I’m just here to…’ and you kind of get bounced from person to person. (*Participant 4*)

*Participant 11* felt that a more proactive stance from healthcare professionals was required:

A lot of women don’t realise it’s going to be difficult or don’t realise they’re not doing it properly, I think there should be a bit more hands on help, people coming round and saying “I’m actually going to check that you’re doing it right,” without waiting for people to ask.

Some women felt that pressure to breastfeed compounded their distress. *Participant 9*, who developed acute pre-eclampsia and experienced a traumatic birth, felt shocked that health professionals would or could not suggest artificial feeding to her overtly:

One of the ward nurses came in and sat down on my bed when I was trying to feed him and said ‘you don’t, I shouldn’t be telling you this, but you don’t have to do this’ and it was such a relief, again to be authorised to not beat myself up about it.

### Relationships in the healthcare system

Women’s relationships with midwives, GPs and health visitors and with the processes surrounding maternity and postnatal care were at the forefront of their described experiences. Many women felt they had been mistreated or ignored. They often associated this with a lack of staff being available, and with the perceived limited staff not having sufficient time to help to the extent mothers felt necessary. Positive experiences were often in the context of developing a supportive relationship with one healthcare provider.

### Uncared for in the healthcare system

Most women spoke of feeling uncared for in the healthcare system at some point during their postnatal period. Most often, women felt that a dialogue with health professionals was missing; that they were not listened to; not asked how they were feeling or not treated as equals in decision-making. *Participant 1* felt that health professionals often did not probe sufficiently to determine whether women were distressed:

Health visitors should be as supportive as they can and *talk* more to people . . . they always seem to refer you on to somebody else, like they don’t want to.

Many experiences of feeling uncared for related to a perceived lack of maternity and neonatal staff, and a perception that time-constrained staff who were available were not approachable, could only deal with major emergencies, or did not fulfil offers of help. *Participant 2* related her experience of hours waiting for help with breastfeeding her new-born:

One of the midwives said to me, “Oh don’t feed your baby, we’ll come in and we’ll help with the breastfeeding”, and like five, six hours later, I’m thinking “Well I’ve got to feed my baby, where are you?”

Once home, women similarly felt that health visitors were often in a rush and did not have time to talk about mothers’ emotions, or did not have sufficient time to assess breastfeeding efficacy. Almost half of the sample perceived that the hospital where they gave birth had made direct errors contributing to their feelings of being uncared for and disrespected. These errors varied greatly, but examples included: being unable to access food or medication whilst catheterised, being sent home before breastfeeding was established or without telling mothers what happened during their labour and birth when complications arose, feeding a baby artificial milk without the mother’s knowledge, not changing blood-stained sheets, being put on inappropriate hospital wards, stitches not being checked resulting in subsequent infections, and, mainly, not being listened to or feeling that staff were unsupportive, as *participant 7* recalled:

The way that I was being talked to during my labour it just made me feel like I didn’t know what I was doing and I should just put it in their hands.

### Unknown in the healthcare system

Beyond feeling directly or indirectly uncared for, many women felt anonymous within the healthcare system. This was characterised by feeling that they did not have one point of contact or one healthcare professional who knew them, their baby and their situation. Women described a ‘tick box’ approach to women’s postnatal wellbeing. This did not facilitate building a relationship within which they could disclose distress. *Participant 1* felt that her depression was not taken seriously at first:

I think the doctors should be more like . . .’cause he just said “Oh you need to talk to the health visitor,” he didn’t seem that interested.

Furthermore, women felt uninformed about sources of support that they could access, and felt that health professionals could do more to link women to local support networks. Consequently there was a sense that mothers could only get support if they were proactive enough to research and access it themselves:

I didn’t think that the care was there easily. I mean there’s a lot of care there if you ask for it, but it isn’t easily accessible. (*Participant 12*)

### Positive experiences

Women did also describe times where they had felt supported within the healthcare system. Almost always this was in the context of having formed a close empathic relationship with one healthcare professional (GP, midwife, health visitor or lactation consultant) within which women felt they could discuss their feelings without being hurried. *Participant 14* commented:

My GP was just, he was understanding, he’s got kids of his own and kids that are quite close together in age and he was telling me about his family, he was very compassionate, understanding, I didn’t feel rushed, um it was, I just got more empathy.

In almost all cases, women experienced these positive relationships through sharing of experiences on the health professional’s part, as *participant 16* recalled after her baby was born prematurely:

There was one particularly great nurse who took good care of the mums . . .but she’d had a premature baby herself, she’d had a baby I think at 33 weeks, the same as [baby] and she really knew what to say to the mums and what to do for the mums, and how to be there for them and to, was really nurturing and really looked after us.

Understanding and unrushed healthcare professionals were viewed as a great help when navigating the new and immediate challenges of motherhood. The immediacy of the challenge is discussed in the next section.

### The shock of the new: diving into motherhood

The term ‘transition’ to motherhood has purposefully been avoided here as women’s reports throughout the interviews were not of transition but more a sudden and challenging change to their life. From being self-sufficient and independent throughout life, many mothers felt vulnerable and dependent for the first time whilst having to learn to manage with a new baby.

### Adjustment to the unknown

A conflict often existed for women, who felt on the one hand that the emotions they were experiencing were ‘normal’ parts of the role of a new mother and were to be expected, yet on the other hand they felt distressed. *Participant 10* had given birth to her third child, but still found it difficult to decide if her distress was ‘normal’:

I was burning myself out by trying to do everything on my own and post-caesarean. Um so it was just really difficult but I kind of didn’t think I had a problem it was kind of like new mums do this all the time and get on with it.

For first time mothers, the lack of a point of reference made it difficult to decide whether feelings of distress were normal, particularly when mothers felt they needed to disentangle tiredness, hormonal changes or feelings of trauma from birth. Sometimes mothers needed to talk to other new or experienced mothers or to a health visitor to decide if they should take some action about their distress. If distress was not encountered as a constant feeling, it could be difficult to decide if action needed to be taken, as *participant 6* described:

It takes quite a while to work out that you do have a problem um and to work out what it is. . .I’ve been . . . Points of terrifying thoughts of having postnatal depression and um then other periods of, you know, thinking I’m absolutely fine.

There was an expectation that life with a baby would be difficult at first but would get easier. Women spoke of the impact of sleep deprivation on their well-being and the sense of eagerly awaiting their baby to sleep for longer stretches so that they could feel better emotionally, as *participant 16* recalled:

Now she’s starting to sleep a little bit more of a stretch of sleep at night …you have more of a normal existence rather than this thing where you’re up all night watching the hours tick by until it’s morning again but you’ve not slept and you just, yeah, you just live in a weird world for a while.

### Overwhelming responsibility

Many women spoke about feeling overwhelmed once their baby was born. Being the responsible adult with total care for their baby left mothers feeling overpowered by a new person that they did not yet know and who demanded so much of them. Sometimes this led to a feeling of wanting to pass the baby over or walk away from their situation, as *participant 16* described:

That’s what makes having a baby hard, is that it’s not something you can give back or to say I’ve had enough of doing this now, it’s not working out, someone else can take over, so um yeah I guess maybe there was the occasional thought of yeah it would be nice to get dressed and walk out the front door and just go out for the day and not ever, or that day think about having a baby.

### Inexperience

Feeling overwhelmed and uncertain was often put in the context of a lack of experience with babies. The new-born period and its challenges were something that was previously inaccessible and that women were not prepared for, having focused on positive aspects throughout their pregnancy. Women tended to feel that being pregnant should have prepared them for being a mother, although *participant 14* described how this was not the real picture:

The whole range of things that are suddenly thrust upon you that you should know, you should know when they need a feed, you should know when they’re thirsty, you *should* know but you don’t, they’re not born with a manual and it’s only through experience and advice that’s passed on…but yet you think ‘I should know, you know, I’ve carried them, they’re mine, I should know what to do.’

Even when women had supportive partners and families, inevitably there came a point when mothers would be at home on their own with the baby. This was a significant time-point for *participant 10*, who highlighted the scary nature of a perceived disconnection between advice about, and reality of, caring for a baby:

You can read as many books as you want but when you get that baby home and you’re kind of on your own you’re like “Right. OK. What do I do now?” Then all the visitors go away and everyone goes back to work and you know then two, three weeks later it’s just you and this baby. It’s very, very scary.

### Meeting new support needs

Women spoke of an increased need for emotional and practical support from partners and close family, as well as a desire to share experiences with others in similar situations.

### Needing and seeking support

Often the relationship women had with their partners was the closest they had and was the only one in which they could disclose everything they were feeling. With the partner usually at work for a large part of the day, there was a build-up of need to talk through how the day with the baby had been, but which had to wait until the partner returned. Women recognised the pressure placed on their partners from working and now supporting them at home with the baby as *participant 7* described:

[Husband]’s having to work really long hours to support us but also coming home and I haven’t been able to do housework . . . he’s just had to have this massive emotional resource for me, and have [baby] and sort the flat out and do all the work so that’s been really affected.

Partners often suggested, encouraged or facilitated accessing additional support. However, even with the most supportive partner, it was sometimes felt that partners simply could not understand what mothers had been through during birth and in looking after the baby all day.

Thus, many women spoke about the importance of accessing support and help outside their relationship with their partner. Whilst it was acknowledged that sometimes professional help was necessary, as a first step there was a sense that mothers had to ensure they got out and met supportive peers. Trying hard to talk to people, to find out what was wrong, and to admit that there was a problem were all considered imperative, if difficult. For example, *participant 10* spoke of a need to persevere with going to postnatal groups to find one that would suit the mother and be source of support:

I think with all new mums, if there’s support there take it. You know it’s very hard to be sort of, you’re ‘I’m a new mum, I’m going to do it all on my own’ but there will come points where being on your own is very, very isolating, very lonely, um, get out there, go to one or two groups you know, you’re not going to know if they’re for you unless you go.

Nonetheless, it was recognised that seeking support was difficult. Women often did not feel like talking when they were feeling particularly distressed. Similarly, admitting to a problem felt like a compromise of their independence and ability to cope. However, when feelings of distress lessened, it was easy to convince oneself that there was not a problem, sometimes resulting in family or close friends demanding that women sought help. Even if women did want to access help, sometimes they felt that their problems were not serious enough to warrant ‘bothering’ health professionals, as *participant 11* recalled when trying to find help with breastfeeding:

The midwives all said ‘Call if you have any problems’ and they did leave a number, but you feel like you’re bothering them, you know, really busy hospital department with people having babies, you don’t really feel like you can phone up and say well I’m having a few problems with breastfeeding.

### Action to help move on

When talking about improvements in how they were feeling, most often women brought up how helpful they had found talking on a one-to-one basis. *Participant 1* felt that if talking did not directly affect the symptoms of distress, social support was appreciated:

Once people realise that I do have, might possibly have, postnatal depression then I’ve started to feel better because they were talking to me, because they were concerned. I didn’t feel better but I had more people to talk to about it.

Some mothers felt more comfortable discussing distress on a one-to-one basis before being comfortable in groups, but many women found that discussing experiences with others in a similar situation was invaluable, ‘to feel like you’re not the only one who’s completely mad having a baby’ (*participant 5*). Groups did not need to be highly-structured or run by health professionals but just needed to include other new mothers, as *participant 2* described:

You also feel like you’re the only one that’s ever had all these problems, then you sit in a group with ten people all having the same problems as you breastfeeding and you think ‘oh’ that it’s not me, you know, it’s not me, it’s not because I’m a bad mum, it’s because that’s life. So that was definitely a help.

However, some mothers felt that they had not found the appropriate place to discuss their feelings. *Participant 6* felt that it would be ‘helpful to be put in touch with other mums in a more similar position’ and *Participant 4* related how she wanted, but felt unable, to discuss her birth experience at a local postnatal group:

The parenting class I went to in [village], everyone had relatively good births, so you kind of feel like the black sheep walking in saying mine was really horrible and I hated it and etcetera etcetera because you feel like a bit of a black cloud to everybody else.

Many mothers felt that beyond talking, the experience of ‘purely getting out of the house’ (*participant 14*) and changing surroundings was important for alleviating distress. *Participant 6* described how vital it was for her to leave the house once a day:

The best thing I did was have a plan for getting out the house every day. That was literally my survival plan so, and it really did work . . .at least you’re out and you don’t feel quite as wracked.

### Parity, birth trauma and previous mental health

It is acknowledged that factors such as parity, mother’s age, previous mental health condition, lower education and traumatic birth are significant predictors of postnatal distress. It was not our intention however to try and explain the reasons for women’s postnatal distress in terms of socio-demographic or maternal factors. Rather, we attempt to illustrate how mothers themselves come to interpret, understand and make sense of their lived experience of distress. Having said that, some interesting patterns emerged within the sample, each of which is described below.

#### Parity

As noted above, six mothers in the sample (35%) had given birth to a second or subsequent baby (articipants 1, 5, 10, 11, 14, 15). Half of the multiparous mothers reported birth-event related distress (10, 11, 15) compared with 8 of 11 (73%) primiparous women. Although multiparous mothers appeared to be less likely than primiparous to report breastfeeding issues (3 of 6 multiparous compared with 9 of 11 primiparous), it is notable that half of the mothers with more than one baby still reported breastfeeding issues. Multiparous mothers appeared less likely to report feelings of detachment or distancing (2 of 6 multiparous compared with 9 of 11 primiparous). Whereas 7 of the 11 primiparous mothers reported feeling stuck or unable to move on, none of the multiparous mothers did. All except one mother with more than one baby (and all primiparas) were represented by the theme ‘The shock of the new’. All multiparous mothers reported positive experiences of the healthcare system (as opposed to 6/11 primiparas), but all women (regardless of parity) reported feeling uncared for *or* anonymous at some point. More multiparous (4 of 6) than primiparous women (5 of 11) were represented in the theme ‘Inexperience,’ which suggests that it is important to consider not only experience with a first child, but also with multiple children. All multiparous women who had a partner spoke of increased need of support from them, compared with 4 of the 11 primiparous women. However, only one multiparous woman described new support needs (compared with 8 of 11 primiparous women). Thus, it appears that in our sample, multiparous and primiparous women comparably endorsed themes, even where it may be expected that one group (primiparas) would endorse a theme such as ‘Inexperience’ more.

#### Traumatic vs not traumatic birth

Eleven mothers (65%) experienced distress related to the labour and birthing experience (articipants 2, 4, 7, 8, 9, 10, 11, 13, 15, 16, 17). Nine of these eleven mothers (82%) spoke of feeling guilty and of blaming themselves, whereas such feelings were reported by only one of the six women who did not experience a traumatic birth. Women who experienced a traumatic birth appeared to have more negative post-natal experiences, they were more likely to speak of feeling distant and detached from their life with their baby (8 of 11 compared with 3 of 6), and more likely to experience difficulties with breastfeeding (9 of 11 compared with 3 of 6). Women who experienced a traumatic birth were more likely to speak of feeling uncared for by the healthcare system (10 of 11 compared with 4 of 6), but a majority in each group spoke of positive experiences of the healthcare system (7 of 11 and 5 of 6). Similar proportions of women who did (6 of 11) and did not experience traumatic births (2 of 6) reported feeling overwhelmed by the responsibility of motherhood, and both groups reported similar support needs.

#### Previous mental health issue

In our sample, seven mothers (41%) disclosed a previous mental health issue (articipants 1, 3, 7, 8, 10, 12, 14). On almost all themes, similar proportions of women with and without a previous mental health issue endorse each theme. For example, five of the seven (71%) women with a previous mental health issue (compared with 6 of 10 other women) described feelings of distance and detachment from their new life with their baby, and four of the 7 women with a previous issue (compared with 5 of 10 other women) described an increased need of support from their partner. However, it is notable that only one of the seven (14%) women with a previous mental health issue spoke of the overwhelming responsibility of motherhood, whereas 7 of 10 (70%) women without a previous issue reported this.

## Discussion

This study explored experiences of postnatal distress phenomenologically by giving priority to the accounts of women interviewed, outside of diagnosed disorders. The results showed the importance to mothers of multiple factors in their experience of becoming distressed and their journey to feeling better. This discussion focuses on three insightful ways in which women explained their distress. First, through temporal points in the process of becoming a mother; distress around birth experiences and establishing and maintaining breastfeeding. Second, through the psychological processes that characterised and maintained distress states, such as feeling overwhelmed, guilt, avoidance and distancing. Third, the importance to mothers of postnatal support: their old, new and changing relationships, for example with healthcare professionals, partners, and other mothers.

### The process of becoming a mother

This study demonstrates the importance of the birth experience to mothers’ subsequent mental health. Birth factors mentioned were largely consistent with previous research which has shown, for example, that low perceived control is associated with low satisfaction with birth, postnatal depression and perceiving the birth as traumatic [33–35]. Furthermore, women in this study corroborated the importance of health-practitioner support in maternal satisfaction with birth, and possibly depression [36, 37]. Quality and continuity of care were also perceived by all participants to be key in their experience of distress around the birth and the early days of caring for their new babies. This is consistent with previous research [18, 38, 39]. This study suggests that research into postnatal distress would benefit from including a measure of how well-supported mothers feel by healthcare professionals, with the aim of understanding the relationship between support during childbirth and multiple types of postnatal distress, beyond PTSD, and in informing development of postnatal care.

Breastfeeding rates in the UK are around the lowest of developed countries (81% breastfeed at least once, but only 42% breastfeed for at least 6 months; [40]). The women in this study wanted to breastfeed but often felt that they had to battle to establish and maintain breastfeeding alone due to the lack of time and lack of practical assistance afforded to helping them in this area by healthcare professionals. Women’s views of having limited support for breastfeeding is consistent with previous research showing that breastfeeding women’s needs for information, practical and emotional support are often unmet due to a lack of health practitioner time and no established relationship with women in need of support [41, 42]. Women did not experience a lack of services to assist with breastfeeding but were frustrated at the lack of clarity over which service would provide the help needed. This fits with the national picture that women in the UK felt less supported with breastfeeding in 2010 than they did in 2005, possibly due to a reduction in the number of health visitors [40]. Of particular importance to women in this study was the all-encompassing nature of feeding problems. With new-born feeding being faced every few hours, the emotions and challenges are compounded, perhaps explaining the definite causal stressor status women attributed to it. It is likely that interventions aimed at resolving breastfeeding difficulties, which could be delivered through primary care, could also resolve emotional difficulties in the mother, as has been the case with other infant-care issues [43].

### Psychological processes

Feelings of being overwhelmed, inexperienced and unsure about their ability to be competent mothers are consistent with previous qualitative research of postnatal depression, anxiety, and posttraumatic stress disorder. A sense of feeling overwhelmed after a traumatic birth merged into feeling a lack of competence at mothering for some women in this study, feelings of guilt, and a struggle to find ways of taking back the mothering role. In this study, breastfeeding was discussed as a way of re-asserting the mother role, a finding observed in other studies [25]. This suggests that breastfeeding could act as a moderator between traumatic birth experience and subsequent distress states. Outside of the traumatic birth literature, a perceived lack of competence in the mothering role contributed to feelings of anxiety, feeling overwhelmed, loneliness [16] and depressive symptoms [23]. Therefore, further research may show that building self-confidence in domains of motherhood could be a valid target for interventions to reduce postnatal distress across disorders.

Mothers’ experiences of avoidance and distancing are also worth further investigation. In a review of coping strategies and maternal well-being, researchers concluded that avoiding focusing on the problematic situation (distancing) is associated with higher odds of developing postnatal depressive symptoms [44]. Avoidance also comprises one of the symptom clusters of PTSD. Mothers in the present study reported avoidance of emotions, thoughts, breastfeeding and of seeking help. Outside the perinatal literature, there is evidence that overt avoidance behaviour is a transdiagnostic process for which behavioural activation (replacing patterns of avoidance with adaptive patterns) has been an effective treatment [28].

### Social support

Researchers have consistently shown that poor communication with healthcare staff and perceived unhelpful staff attitudes are detrimental to new mothers [39, 45–47]. The women in this study largely corroborated the negative effect on their wellbeing of feeling ignored, uncared for, or poorly communicated with during birth, the postnatal hospital stay, postnatal home-care and concerning breastfeeding. Conversely, women in this study described a positive effect of the presence of healthcare staff who were parents themselves, who were experienced in the challenges of motherhood and who were able to provide high levels of empathy. This supports previous research showing that feeling ‘mothered’, cared for and listened to helps new mothers to feel confident and well-recovered from birth [42, 48]. Regarding in-patient care, Brown et al. [45] found that the sensitivity and understanding in interactions with caregivers had the greatest effect on women’s ratings of care. The findings of this study confirm that listening and communicating skills of postnatal staff are an important area for development [39]. The well-voiced view that postnatal services were understaffed and the staff working were perceived as too busy was also apparent for women in this study [41]. A key issue in perceptions of care is how individual healthcare providers interact with and listen to women [39, 42]. In line with research showing that multiple psychological approaches to intervention can be beneficial to a mother’s mental health (e.g., for PTSD [49]; for postnatal depression, [50]) it may be that training in listening and counselling skills combined with time to practise these skills with new mothers may be enough to reduce levels of distress.

Social support from a partner is well-documented as being a protective factor against depressive symptoms [44]. Women in this study spoke of increased demands on their partner but felt that these were largely met and that support was lacking in other areas, primarily from health professionals. Furthermore, the voiced need of mothers to connect with and speak to others in a similar situation was universal. Peer support has led to fewer depressive symptoms in high-risk mothers and this type of support may be useful across different types of distress [51].

### Parity, birth trauma and previous mental health

Clear differences between multiparous and primiparous women were not seen in experiences of distress in this sample. This largely reflects the picture from quantitative studies which shows that the relationship between parity and postnatal distress is unclear. In a sample of 5252 Danish mothers of whom 5.5% were depressed at 4 months postpartum, previous psychiatric illness and high parity were important risk factors for developing postnatal depression [52], whereas in 944 Swiss mothers experiencing distress in the early postpartum days, primiparity was a risk factor for maternal distress [53].

Concerning birth trauma, it is estimated that up to 30% of women experience subclinical debilitating symptoms of posttraumatic stress disorder after birth [54, 55]. Our study was not diagnostic in nature, many mothers reported feeling debilitated by their birth experience. There is also evidence that anxiety and symptoms of posttraumatic stress following childbirth are strong predictors of postnatal depression [8, 56].

Previous psychiatric illness is a risk factor for postnatal distress that has been confirmed in multiple large cohort studies [8, 52] and seven mothers in our sample disclosed a previous mental health issue. However, these women experience of distress did not qualitatively differ in our sample. Further longitudinal qualitative research to examine the experiences of women who are not distressed antenatally and follow them into the postpartum period would help to address this question.

### Limitations

We do not claim that these findings apply to all mothers who have experienced postnatal distress. The sample was self-selected and it is probable that participants were motivated to talk about their experiences and take action. The women who took part were mostly white, well-educated, employed before having their baby, and in long-term relationships. Further exploration with women of different ethnicities, relationship status, and ages would be beneficial to expand on and compare with the findings in our study. It could be considered that using a broad definition of distress (‘any emotional difficulties’) could lead to inclusion of women with normal levels of adjustment difficulties that would dissipate over time. However, all the women in this study experienced distress that they felt they needed extra support with, regardless of diagnosis.

## Conclusions

This study has addressed two important issues that need to be explored in order to improve understanding of postnatal distress. The first is how women experience and make sense of their distress. Women clearly explained their emotional difficulties as relating to both key temporal points in the process of becoming a mother (particularly childbirth and breastfeeding) and in terms of psychological processes across different types of distress (particularly feelings of detachment, avoidance, guilt and social comparison). The second issue concerns the aspects of distress mothers felt they needed help with and how they experienced that help. Breastfeeding again emerged as a key area with which women felt they needed support with but which was not easily available. Mothers also felt that they needed support with resolving feelings around traumatic births. A desire to validate and normalise feelings through talking both in groups and on a one-to-one basis with healthcare providers such as midwives or health visitors was universal.
